# Programmed Cell Death Ligand 1 Is Essential for Electroacupuncture-Mediated Analgesia in the Cerebellum of Fibromyalgia Mice

**DOI:** 10.3390/biomedicines14030584

**Published:** 2026-03-05

**Authors:** Hung-Yu Huang, Younbyoung Chae, Ming-Chia Lin, I-Han Hsiao, Hsin-Cheng Hsu, Chien-Yi Ho, Yi-Wen Lin

**Affiliations:** 1 Graduate Institute of Acupuncture Science, College of Chinese Medicine, China Medical University, Taichung 404328, Taiwan; 114304001@365.cmu.edu.tw; 2 Department of Neurology, China Medical University Hospital, Taichung 404327, Taiwan; 3 Acupuncture and Meridian Science Research Center, Kyung Hee University, Seoul 02453, Republic of Korea; ybchae@khu.ac.kr; 4 Department of Nuclear Medicine, E-DA Hospital, I-Shou University, Kaohsiung 82445, Taiwan; ed101186@edah.org.tw; 5 School of Medicine, College of Medicine, China Medical University, Taichung 404328, Taiwan; 018309@tool.caaumed.org.tw; 6 Department of Neurosurgery, China Medical University Hospital, Taichung 404327, Taiwan; 7 Department of Traditional Chinese Medicine, China Medical University Hsinchu Hospital, Hsinchu 302233, Taiwan; 002762@tool.caaumed.org.tw; 8 Department of Biomedical Imaging and Radiological Science, China Medical University, Taichung 404328, Taiwan; peishiuan1010@gmail.com; 9 Division of Family Medicine, China Medical University Hsinchu Hospital, Hsinchu 302233, Taiwan; 10 Physical Examination Center, China Medical University Hsinchu Hospital, Hsinchu 302233, Taiwan; 11 Department of Medical Research, China Medical University Hsinchu Hospital, Hsinchu 302233, Taiwan; 12 Chinese Medicine Research Center, China Medical University, Taichung 404328, Taiwan

**Keywords:** fibromyalgia, electroacupuncture, PD-L1, *Trpv1*, neutralization, cerebellum

## Abstract

**Background:** Fibromyalgia is a chronic disease that predominantly affects women and lasts over several months, causing problems both for individuals and society. While several studies have demonstrated the potential of electroacupuncture (EA) to alleviate fibromyalgia pain in mice, further research is needed to investigate its underlying mechanisms. Programmed cell death ligand 1 (PD-L1)/PD-1 were first identified to be involved in cancer immunotherapy, and their application to pain management has not been yet investigated. **Methods:** In this study, we aimed to explore the mechanism underlying the action of PD-L1 on the PD-1 pathway in a mouse model of fibromyalgia. **Results:** We established such a mouse model using intermittent cold stress (ICS) and confirmed mechanical (D4: 2.02 ± 0.13 g, *n* = 9) and thermal (D4: 4.28 ± 0.21 s, *n* = 9) hyperalgesia. We found that EA, intracerebral ventricle (ICV) PD-L1 injection, and *transient receptor potential vanilloid 1* (*Trpv1*) knockout effectively counteracted hyperalgesia. We observed low PD-1 expression in the cerebellum of fibromyalgia mice but increased expression of TRPV1 and pain-related kinases. These phenomena could be further reversed by EA, ICV PD-L1 injection, and *Trpv1* knockout. To confirm that these effects were caused by PD-L1 release, we added PD-L1-neutralizing antibodies to the EA and PD-L1 treatment. The analgesic effects and EA and PD-L1 mechanisms were inhibited. **Conclusions:** Our results elucidate the role of the PD-L1/PD-1 pathway in EA treatment of fibromyalgia and reveal its potential value for fibromyalgia management.

## 1. Introduction

Programmed cell death protein 1 (PD-1), first discovered in 1992, is a membrane protein expressed on T cells and primarily involved in apoptosis. Its structure is mainly composed of 288 amino acids, including an N-terminal domain. PD-1 is also present on the cell membranes of T lymphocytes, B lymphocytes, and natural killer cells [1]. Recent reports indicate that the main function of PD-1 is regulating T cell activity in tissues to control cancer cells. Cancer cells can suppress the immune system, making T cells unable to kill them. This process is mediated by PD-1. When this protein is expressed on the surface of T cells, the PD-L1 expressed on the surface of cancer cells binds to and inhibits it [2]. In acute and chronic pain models, PD-L1 and PD-1 binding is involved in pain relief [3]. Therefore, studying the PD-L1/PD-1 pathway and its related pathways is highly important in fibromyalgia (FM) models.

Clinical diagnosis of FM currently lacks standardization, relying primarily on physicians’ subjective judgment. While numerous questionnaires can help assess the symptoms and severity of FM, scientifically validated data are still lacking. FM is a chronic pain disorder affecting numerous aspects of living, including personal, healthcare, and economic elements. It is more common in women and frequently accompanied by tension headaches, irritable bowel syndrome, anxiety, and depression. Women generally have a significantly lower pain threshold than men and experience more severe symptoms [4]. Currently, there is no effective cure for FM; medications can only relieve symptoms. Novel therapeutic strategies for FM involve the use of specific circulating microRNAs [5]. Nonpharmacological options, such as noninvasive brain stimulation, exercise, yoga, acupuncture, and nutritional therapy, are being explored to alleviate symptoms and improve quality of life [6]. FM is caused by repeated central nervous system activation, in what is known as central sensitization. Its incidence is about 2–8%, and its etiology and pathogenesis are not fully understood. Recent studies have indicated that inflammatory cytokines may damage neural circuits, leading to central sensitization and neuroinflammation [7].

Acupuncture has been used in Asia for over 3000 years, and recently, it has been optimized to treat many diseases. The WHO recognizes acupuncture as applicable to over 100 diseases, particularly for pain management. It is a method for balancing the flow of Qi and blood and promoting the health of the body’s meridians. Western medicine has accepted that acupuncture can relieve symptoms by stimulating peripheral nerves, connective tissues, and muscles [8]. Particularly, electroacupuncture (EA) could enhance anti-inflammatory effects and effectively control inflammation in a mouse sepsis model [9]. Recent studies reported that EA can effectively stimulate the vagal–adrenal axis in mice by stimulating the ST36 acupoint [10]. Tissue damage can lead to the activation of glial cells and immune cells in the nervous system, producing excessive pro-inflammatory cytokines, which in turn cause chronic pain. In addition, inflammation of the central nervous system can drive central sensitization, affecting neural circuits and promoting the occurrence of chronic pain [11,12]. Consistently with this, our previous research showed that EA can release adenosine triphosphate, interleukin 1β (IL-1β), and IL-6 at local acupoints [13]. Further evidence suggests that EA can treat inflammatory pain [14,15,16], neuropathic pain [17,18], and fibromyalgia [19,20,21,22] in animals and medicine. Neuroinflammation plays an increasingly important role in the brain and is associated with many diseases [23]. In addition, EA can treat pain and depression comorbidities by reducing inflammatory factors in plasma such as ILs, tumor necrosis factor-alpha (TNF-α), and interferon gamma (IFN-γ) [24].

Based on the above, we wanted to investigate whether EA can treat mechanical and thermal hyperalgesia caused by intermittent cold stress (ICS)-induced FM pain, whether EA analgesia is related to PD-L1/PD-1, and the effect of PD-L1/PD-1 on the TRPV1 signaling pathway. First, we established a mouse FM model using ICS, which successfully induced mechanical and thermal hyperalgesia in mice, which EA could alleviate; it could reverse the significant changes observed in the expression levels of PD-L1/PD1 and related molecules in the mouse cerebellum. Similar results were found in mice receiving PD-L1 injection or with *Trpv1* deletion. To confirm that EA can relieve pain by increasing PD-L1, we administered PD-L1-neutralizing antibodies during EA and ICV PD-L1 injection; both the analgesic effect and the involved molecular mechanisms were attenuated. Therefore, our findings suggest that the analgesic effect of EA may be related to TRPV1 signaling regulation by PD-L1/PD1 in the mouse cerebellum. Accordingly, we propose that EA can modulate PD-L1/PD1 signaling, being useful for treating FM.

## 2. Materials and Methods

### 2.1. Animals and Fibromyalgia Pain Induction

Our mouse experiments were approved by the Institute of Animal Care and Use Committee of China Medical University (Permit no. CMUIACUC-2024-076), Taiwan, following the Guide for the use of Laboratory Animals (National Academy Press). We selected 8–12-week-old female C57B/L6 wild-type mice, purchased from BioLasc Taiwan Ltd. (Yilan, Taiwan), weighing approximately 18–22 g. The animals were directly transported and housed in a specific pathogen-free environment; all mice had healthy coats and no wounds. Upon arrival, the mice were immediately placed in an environment under a 12 h light/dark cycle (6:00 a.m. to 6:00 p.m.), at a room temperature of 25 °C and a humidity of ~60%. To estimate the sample size requirements, we used the G*Power 3.1.9.7 program; each group contained nine mice to achieve a significance level of α = 0.05 and a statistical power of 80%. The mice were randomly assigned to five groups: a normal group (normal); a cold stress-induced FM group (FM); FM mice treated with 2 Hz EA (FM + 2 Hz EA); FM mice treated with intraventricular PD-L1 injection (FM + PD-L1); and *Trpv1*^−/−^ mice with FM induction (FM + *Trpv1*^−/−^). To initiate FM pain, mice were placed in a 4 °C environment, while the normal group was maintained at a constant temperature of 25 °C. At 10 a.m. the following day, these FM mice were moved back to 25 °C for 30 min, before being transferred back to 4 °C for 30 min. This process was continued for 6 h until 4 p.m., before they were repositioned again overnight from 4 p.m., over the first three days.

### 2.2. Electroacupuncture

For EA, mice were anesthetized with 5% isoflurane for induction and 1% isoflurane for maintenance. Two 1-inch stainless steel needles (32G, Yu Kuang Chem. Ind. Corp., New Taipei city, Taiwan) were inserted perpendicularly to the ST36 acupoint on both sides. The mouse ST36 is located 3–4 mm under the patella, between the fibula and tibia, and on the anterior side of the anterior tibial muscle. EA was performed using an electronic Trio 300 stimulator (Ito, Tokyo, Japan) with continuous square wave pulses of a 1 mA intensity, 2 Hz frequency, and 150 μs pulse width for 20 min. During electrostimulation, slight twitching occurred in the muscles around the acupoint. EA was performed twice on days 3 and 4 after FM induction.

### 2.3. Nociceptive Behavior Measurements

We first placed each mouse in an acrylic box above a wire mesh to facilitate pain testing. The mice were kept in a dark, noise-free, room-temperature environment to allow them to calm down and acclimatize for 30 min. Testing was conducted while the mice were inactive, not standing, not sleeping, not scratching, and not grooming. Mechanical and thermal pain were measured on days 0, 3, and 4 before and after FM induction, with a total of three measurements. The pain threshold before ICS induction was used as a baseline. We used electronic von Frey filaments for all tests, 10 min apart (IITC Life Science Inc., Woodland Hills, CA, USA). We then used Hargreaves’ test to measure the thermal withdrawal latency of the mouse paw to a radiant heat source using an IITC plantar analgesia device (SERIES8, Model 390G, IITC Life Science Inc., Woodland Hills, CA, USA). This device has a 20 s automatic power-off function to avoid injury to the mouse’s hind paw.

### 2.4. Western Blot Analysis

The mouse cerebellum was dissected for protein extraction, before which tissues were placed on ice and stored at −80 °C. Entire proteins were isolated in cold radioimmunoprecipitation (RIPA) lysis buffer containing 50 mM Tris-HCl at a pH of 7.4, 250 mM NaCl, 1% NP-40, 5 mM EDTA, 50 mM NaF, 1 mM Na3VO4, 0.02% NaN3, and 1× protease inhibitor cocktail (AMRESCO). The extracted proteins were subjected to 8% SDS-Tris glycine gel electrophoresis and transmitted to a polyvinylidene difluoride (PVDF) membrane incubated first in 5% non-fat milk in TBS-T buffer (10 mM Tris at a pH of 7.5, 100 mM NaCl, 0.1% Tween 20) before incubation with a primary antibody in TBS-T with 1% bovine serum albumin (BSA) for 1 h at room temperature. Peroxidase-conjugated antirabbit or antimouse antibody (1: 5000) was used as the secondary antibody. Blot bands were imagined using a chemiluminescent substrate kit (PIERCE, Appleton, WI, USA) and LAS-3000 Fujifilm (Fuji Photo Film Co., Ltd., Tokyo, Japan). Where relevant, the protein concentration of the bands was measured using NIH Image J 1.54h software (Bethesda, MD, USA). β-actin or α-tubulin was used as the internal control.

### 2.5. Immunofluorescence

The mice were euthanized with a 5% isoflurane and intracardially injected with 0.9% normal saline followed by 4% paraformaldehyde. Tissues were instantly excised and fixed with 4% paraformaldehyde at 4 °C for three days. Then, samples were embedded in 30% sucrose for cryoprotection overnight at 4 °C. Next, the tissues were fixed in an optimal-cutting-temperature complex and quickly frozen in liquid nitrogen before storage at −80 °C. The frozen tissues were cut into 20 mm sections using a cryostat directly placed on glass slides. The sections were fixed with 4% paraformaldehyde and incubated with a blocking solution consisting of 3% BSA, 0.1% Triton X-100, and 0.02% sodium azide for 1 h at room temperature. Then, the samples were incubated overnight with the primary antibody (1:200, Alomone, Jerusalem, Israel) for PD-L1 and TRPV1 in 1% BSA solution. Next, the samples were incubated with the secondary antibody (1:500), 488-conjugated AffiniPure donkey antirabbit IgG (H + L) or 594-conjugated AffiniPure donkey antigoat IgG (H + L), for 2 h at room temperature before fixation with cover slips for immunofluorescence visualization.

### 2.6. Intracerebroventricular Injection

The mice were maintained under 1% isoflurane anesthesia with their heads fixed in a stereotaxic apparatus. A cannula, a 23-gauge 2 mm stainless steel tube, was inserted into the ventricle, secured, and placed subcortically at 0.5 mm along the anteroposterior axis, around 1 mm along the mediolateral axis, and about 2.5 mm along the dorsoventral axis, and it was fixed to the skull with dental cement. After insertion into the ventricle, it was connected to a Hamilton syringe with a PE tube (PE10, Portex, Kent, UK). Using an injection pump (KD Scientific, Shanghai, China), 5 μg of PD-L1- or anti-PD-L1-neutralizing Ab (PD-L1 Ab; Sigma, St. Louis, MO, USA) (2 μL/ventricle) was injected into the ventricle over 5 min. After injection, the cannula was maintained in the ventricle for 2 min to allow for continued PD-L1- or anti-PD-L1-neutralizing antibody diffusion.

### 2.7. Statistical Analysis

Statistical analysis was performed using the SPSS 21.0 statistic program. All statistical data are presented as the mean ± standard error of the mean. Differences among all groups were tested using ANOVA, followed by a post hoc Tukey test. *p* < 0.05 was considered to indicate statistical significance. The experimental researchers were blinded in group allocation and data analysis. To prevent imbalances during the study, mice were randomly assigned to the required groups.

## 3. Results

### 3.1. Intermittent Cold Stress Successfully Induced Mechanical and Thermal Hyperalgesia Alleviated by EA, ICV PD-L1, and Trpv1 Deletion

**Figure 1:**
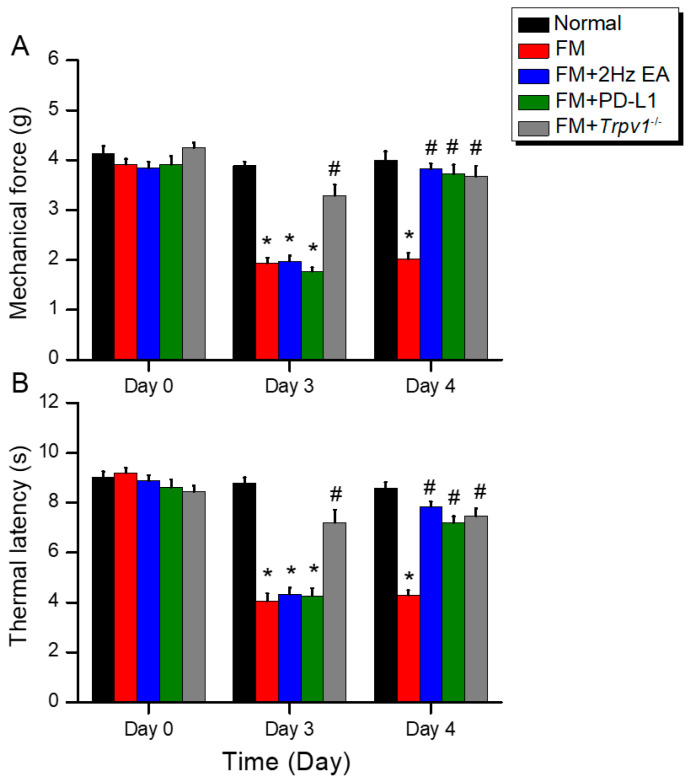
Diagram characterizing mechanical and thermal hyperalgesia in mice. (**A**) von Frey filament test indicating the mechanical threshold and (**B**) Hargreaves’ test for thermal latency. * *p* < 0.05 vs. normal group. ^#^ *p* < 0.05 vs. FM group. *n* = 9 per group. A one-way ANOVA test was used to determine the differences between the groups, followed by a post hoc Tukey test.

To assess the functional mechanisms underlying FM, we first established a mouse model of FM using ICS. In wild-type mice, the von Frey test showed normal mechanical pain thresholds at baseline (Figure 1A, black column, day 0: 4.13 ± 0.16 g, *n* = 9). On days 3 (Figure 1A, red column, day 3: 1.93 ± 0.12 g, *n* = 9) and 4 (Figure 1A, red column, day 4: 2.02 ± 0.13 g, *n* = 9) in FM mice, we observed significant mechanical hypersensitivity. Administering EA (Figure 1A, blue column, day 4: 3.83 ± 0.11 g, *n* = 9) or ICV PD-L1 injection (Figure 1A, green column, day 4: 3.72 ± 0.19 g, *n* = 9) to FM mice resulted in strong analgesic effects. Similar results were observed in *Trpv1*^−/−^ mice (Figure 1A, gray column, day 4: 3.69 ± 0.21 g, *n* = 9). We further used Hargreaves’ test to examine thermal hypersensitivity. All groups had normal thermal pain thresholds. On days 3 (Figure 1B, red column, day 3: 4.04 ± 0.32 s, *n* = 9) and 4 (Figure 1B, red column, day 4: 4.28 ± 0.21 s, *n* = 9), FM mice exhibited thermal hyperalgesia, which persisted for two days. Notably, EA (Figure 1B, blue column, day 4: 7.86 ± 0.20 s, *n* = 9) and ICV PD-L1 injection (Figure 1B, green column, day 4: 7.19 ± 0.26 s, *n* = 9) reduced pain hypersensitivity on day 4. To explore the relationship between PD-L1 and TRPV1 in FM, we also induced ICS in *Trpv1*^−/−^ mice. However, thermal hypersensitivity was not induced in *Trpv1*^−/−^ mice on day 4 (Figure 1B, gray column, day 4: 7.48 ± 0.29 s, *n* = 9).

### 3.2. PD-1 Was Attenuated and Nociceptive TRPV1 Signaling Increased in the CB5 Region of FM Mice, Phenomena Reversed by EA, ICV PD-L1 Injection, and Trpv1 Deletion

**Figure 2:**
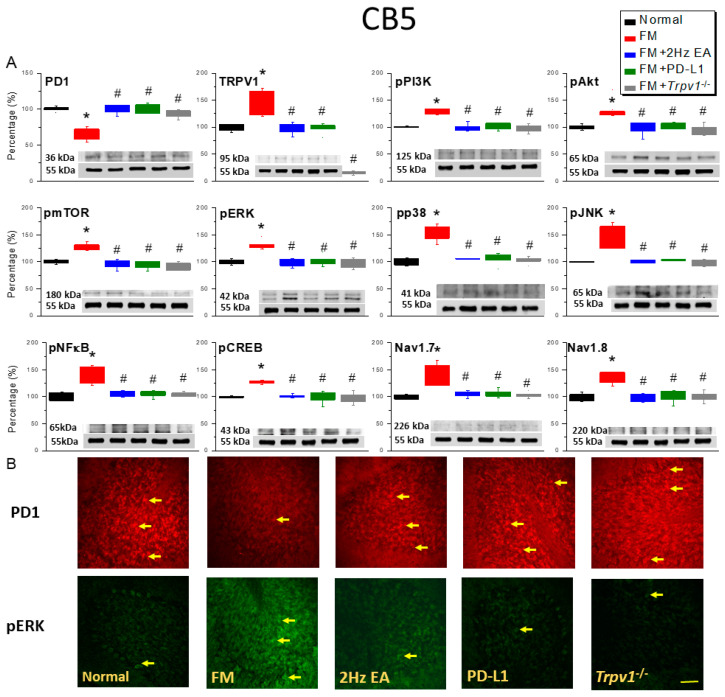
EA, ICV PD-L1 injection, and *Trpv1*^−/−^ increased PD-1 and decreased TRPV1 pathway expression in the CB5 region of FM mice. (**A**) Western blot indicates that EA, PD-L1, and *Trpv1*^−/−^ noticeably increased PD-1 and decreased TRPV1 pathway expression. (**B**) Immunofluorescence staining for PD-1 (red) and pERK (green) in fibromyalgia mice. Yellow arrows indicate immune-positive signals. Bar = 100 μm. * *p* < 0.05 vs. normal group. ^#^ *p* < 0.05 vs. FM group. FM = fibromyalgia (per group, *n* = 6 in Western blot and *n* = 3 in immunofluorescence staining). A one-way ANOVA test was used to determine the differences between the groups, followed by a post hoc Tukey test.

We first investigated PD-1 expression in the CB5 region of mice with ICS-induced FM. Western blot results showed that wild-type mice had normal PD-1 protein levels in this region (Figure 2A, black column, day 4: 100.00 ± 1.2%, *n* = 6), while ICS-induced FM mice showed a significant reduction in expression (Figure 2A, red column, day 4: 66.25 ± 3.53%, *n* = 6). EA (Figure 2A, blue column, day 4: 99.59 ± 2.59%, *n* = 6) and ICV PD-L1 (Figure 2A, green column, day 4: 99.89 ± 2.81%, *n* = 6) injection significantly increased PD-1 protein levels in the CB5 region, as observed in *Trpv1*^−/−^ mice (Figure 2A, gray column, day 4: 93.62 ± 2.26%, *n* = 6). Furthermore, on day 4 after ICS-induced FM pain, TRPV1 concentration in the CB5 region of FM mice was significantly increased (Figure 2A, black column, day 4: 140.99 ± 9.20%, *n* = 6). EA (Figure 2A, blue column, day 4: 97.90 ± 4.29%, *n* = 6) and ICV PD-1 injection (Figure 2A, green column, day 4: 98.81 ± 3.77%, *n* = 6) inhibited TRPV1 overexpression in this region; in mice with *Trpv1* deletion, TRPV1 protein almost disappeared. Next, we observed a significant increase in the levels of proteins involved in downstream pPI3K/pAkt/pmTOR pathways (Figure 2A, black columns, *n* = 6). Similarly, EA (Figure 2A, blue columns, *n* = 6), ICV PD-1 injection (Figure 2A, green columns, *n* = 6), and *Trpv1* deletion (Figure 2A, gray columns, *n* = 6) effectively inhibited overexpression of pPI3K/pAkt/pmTOR signaling mediators. Compared with normal mice, FM mice showed a relative increase in functional phosphorylation of pERK, pp38, and pJNK (Figure 2A, red columns, *n* = 6). In ICS-induced EA (Figure 2A, blue columns, *n* = 6) and ICV-injected PD-L1 mice (Figure 2A, green columns, *n* = 6), phosphorylation was reduced, while *Trpv1*^−/−^ mice showed normal pERK, pp38, and pJNK levels after ICS induction (Figure 2A, gray columns, *n* = 6).

Next, we moved our focus to transcription factors; Western blot results showed normal pNFκB and pCREB expression in the CB5 region of wild-type mice and significantly increased expression in FM mice (Figure 2A, red columns, *n* = 6). In the EA (Figure 2A, blue columns, *n* = 6) and PD-L1 groups (Figure 2A, green columns, *n* = 6), pNFκB and pCREB expression was significantly reduced, similarly to what was observed in *Trpv1*^−/−^ mice (Figure 2A, gray columns, *n* = 6). We further investigated the possible role of the Nav1.7 and Nav1.8 nociceptive channels. Our data indicated higher activity of both these ion channels after induction (Figure 2A, red columns, *n* = 6). In the EA (Figure 2A, blue columns, *n* = 6), PD-L1 (Figure 2A, green columns, *n* = 6), and *Trpv1*^−/−^ groups, overexpression was reduced. Immunofluorescence staining showed normal PD-1 expression in the CB5 region, which decreased after FM induction (Figure 2B, *n* = 3). In the EA, PD-L1, and *Trpv1*^−/−^ groups (Figure 2B, *n* = 3), the number of PD-1 immune granules in the CB5 cell layer increased. Conversely, the quantity of pERK was normal, but this was enhanced in FM mice. pERK overexpression was attenuated in mice stimulated with EA and PD-L1, as well as in *Trpv1*^−/−^ mice (Figure 2B, *n* = 3).

### 3.3. Effects of EA, ICV PD-L1 Injection, and Trpv1 Deletion on Cold Stress-Induced Fibromyalgia Pain in the CB6 and CB7 Regions

**Figure 3:**
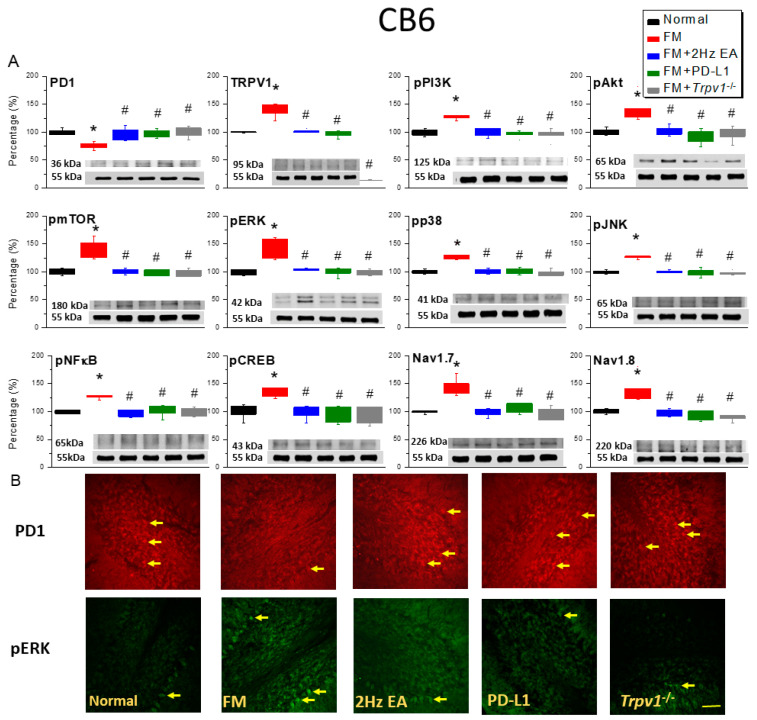
Low PD-1 expression in the CB6 region of ICS-induced FM mice. (**A**) Relative PD-1 and TRPV1 pathway expression in all groups, as indicated by Western blot analysis. (**B**) Immunofluorescence for PD-1 (red) and pERK (green) expression in five experimental groups. Yellow arrows indicate immune-positive signals. Bar = 100 μm. * *p* < 0.05 vs. normal group. ^#^ *p* < 0.05 vs. FM group. All results are representative of ≥6 independent experiments for Western blot and three for immunofluorescence staining. A one-way ANOVA test was used to determine the differences between the groups, followed by a post hoc Tukey test.

**Figure 4:**
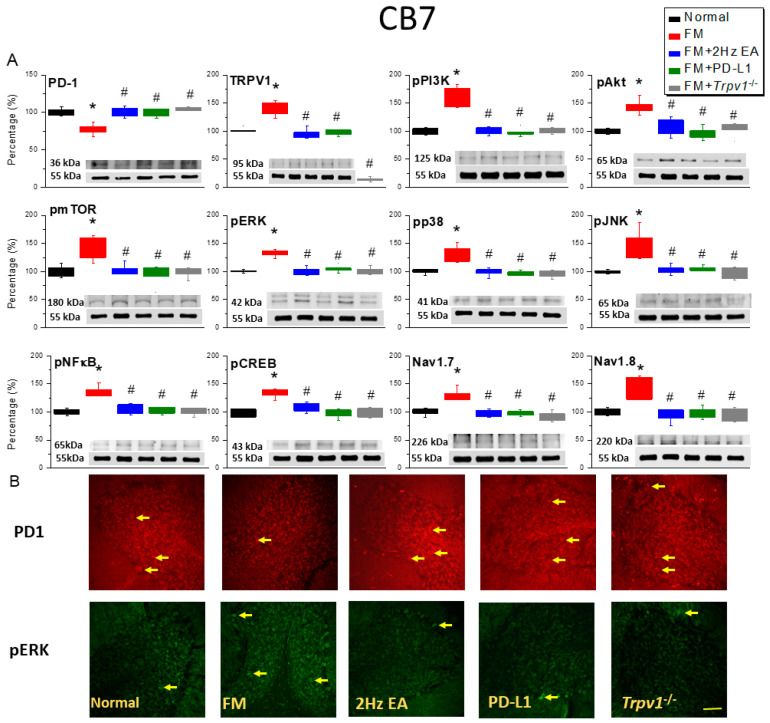
PD-1/TRPV1 interaction is responsible for nociception of ICS-induced FM pain. Protein concentration of PD-1- and TRPV1-related molecules in the CB7 area of mice. Western blotting contained five lanes: normal, FM, FM + EA, FM + PD-L1, and FM + *Trpv1*^−/−^. (**A**) PD-1- and TRPV1-associated protein expression. (**B**) Immunofluorescence for PD-1 (red) and pERK (green) expression in all groups. Yellow arrows indicate immune-positive signals. Bar = 100 μm. * *p* < 0.05 vs. normal group. ^#^ *p* < 0.05 vs. FM group. *n* = 6 independent experiments for Western blot and *n* = 3 for immunofluorescence staining. A one-way ANOVA test was used to determine the differences between the groups, followed by a post hoc Tukey test.

Next, we evaluated PD-1 expression in the CB6 and CB7 regions after inducing FM. In a Western blot, PD-1 content in both regions of FM mice relatively decreased compared to that in normal mice (CB6: Figure 3A, red column, day 4, 75.49 ± 2.35%, *n* = 6; CB7: Figure 4A, red column, day 4, 82.76 ± 3.56%, *n* = 6), and EA (CB6: Figure 3A, blue column, day 4, 98.18 ± 4.19%, *n* = 6; CB7: Figure 4A, blue column, day 4, 101.11 ± 2.61%, *n* = 6) and ICV PD-1 injection (CB6: Figure 3A, green column, day 4, 98.45 ± 2.77%, *n* = 6; CB7: Figure 4A, green column, day 4, 99.77 ± 2.12%, *n* = 6) increased this in the CB6 and CB7 regions. Compared with those in the FM group, the PD-1 receptors in *Trpv1*^−/−^ mice reached similar levels to those in the normal group on day 4 after FM induction (CB6: Figure 3A, gray column, day 4, 100.23 ± 3.82%, *n* = 6; CB7: Figure 4A, gray column, day 4, 102.73 ± 2.43%, *n* = 6). We then investigated the effect of PD-1 on TRPV1. Compared with that in normal mice, TRPV1 expression in the CB6 and CB7 regions of FM mice significantly increased (CB6: Figure 3A, red column, day 4, 138.17 ± 4.57%, *n* = 6; CB7: Figure 4A, red column, day 4, 139.76 ± 4.85%, *n* = 6), an effect attenuated by EA (CB6: Figure 3A, blue column, day 4, 99.25 ± 2.80%, *n* = 6; CB7: Figure 4A, blue column, day 4, 96.18 ± 3.28%, *n* = 6) and ICV PD-L1 injection (CB6: Figure 3A, green column, day 4, 97.65 ± 2.27%, *n* = 6; CB7: Figure 4A, red column, day 4, 97.73 ± 2.04%, *n* = 6). As expected, almost no TRPV1 protein expression was observed in *Trpv1*^−/−^ mice. Another Western blot showed significant overexpression of downstream TRPV1 molecules (such as pPI3K/pAkt/pmTOR) in the CB6 (Figure 3A, red column, *n* = 6) and CB7 (Figure 4A, red column, *n* = 6) regions of FM mice. Conversely, significant pPI3K/pAkt/pmTOR inhibition was observed in the EA and PD-L1 treatment groups (Figure 3A and Figure 4A, blue and green columns, *n* = 6). Compared with that in FM mice, pPI3K/pAkt/pmTOR signaling was not enhanced in CB6 and 7 *Trpv1*^−/−^ mice (Figure 3A and Figure 4A, gray columns, *n* = 6).

We then explored the involvement of pERK, pp38, and pJNK in the CB6 and CB7 regions of FM mice. Their expression was significantly increased in FM mice (Figure 3A and Figure 4A, red columns, *n* = 6) and attenuated in mice treated with EA (Figure 3A and Figure 4A, blue columns, *n* = 6) and ICV PD-L1 injection (Figure 3A and Figure 4A, green columns, *n* = 6), as well as in the *Trpv1*^−/−^ group (Figure 3A and Figure 4A, gray columns, *n* = 6). Similarly, pNFκB and pCREB expression significantly increased in the CB6 and CB7 regions of FM mice (Figure 3A and Figure 4A, red columns, *n* = 6), an effect mitigated in mice treated with EA and ICV PD-L1 injection, as well as in the *Trpv1*^−/−^ group (Figure 3A and Figure 4A, blue, green, and gray columns, *n* = 6). Similar results were obtained for Nav1.7 and Nav1.8 pain receptor channels. Immunofluorescence staining showed PD-1-positive cells in the CB6 and CB7 regions of normal mice, but these cells were significantly reduced in FM mice. EA, ICV PD-L1 injection, and *Trpv1* deletion restored the number of PD-1-positive cells (Figure 3B and Figure 4B, *n* = 3). Further, qualitative analysis showed a significant increase in the number of pERK-positive cells in the CB6 and CB7 regions of FM mice, similar to the trend observed in the Western blot. In contrast, the number of pERK-positive cells decreased in mice treated with EA, ICV PD-L1 injection, and *Trpv1* deletion (Figure 3B and Figure 4B, *n* = 3).

### 3.4. ICV PD-L1-Neutralizing Antibodies Reversed the Analgesic Effects of EA and PD-L1 in Fibromyalgia Mice

**Figure 5:**
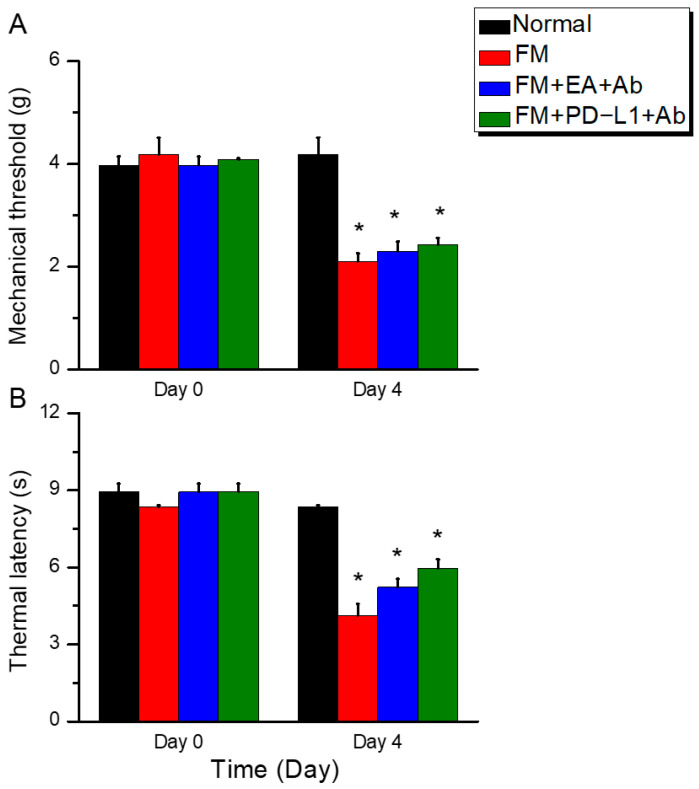
Administration of a PD-L1-neutralizing antibody attenuated 2Hz EA, PD-L1, and *Trpv1*^−/−^ analgesia. (**A**) von Frey filament test indicating the mechanical threshold. (**B**) Hargreaves’ test for thermal latency. * *p* < 0.05 vs. normal group. *n* = 9 per group. A one-way ANOVA test was used to determine the differences between the groups, followed by a post hoc Tukey test.

In normal mice, regular mechanical and thermal pain thresholds were observed on days 0 and 4 (Figure 5A, black column: day 0: 3.98 ± 0.17 g; day 4: 4.19 ± 0.33 g; *n* = 9). Further results showed that mice receiving the ICS model exhibited significant mechanical (Figure 5A, red column, day 4: 2.10 ± 0.16 g, *n* = 9) and thermal hyperalgesia (Figure 5B, red column, day 4: 4.13 ± 0.46 s, *n* = 9). After ICV injection of PD-1-neutralizing antibodies, the analgesic effect of EA on both mechanical or thermal hyperalgesia significantly decreased, indicating that PD-L1 plays a crucial role in EA analgesia (Figure 5A, blue column: day 0: 3.98 ± 0.17 s; day 4: 4.19 ± 0.33 g; *n* = 9; Figure 5B, blue column: day 4: 5.23 ± 0.32 s; *n* = 9). Similarly, on day 4 after ICS induction, the analgesic effect of ICV PD-1 injection on mechanical and thermal pain diminished due to the PD-L1-neutralizing antibodies (Figure 5A, green column, day 4: 2.43 ± 0.14 g, *n* = 9; Figure 5B, green column, day 4: 5.95 ± 0.37 s, *n* = 9).

### 3.5. The Analgesic Effect of EA and PD-L1 Injection Was Reversed by PD-L1-Neutralizing Antibody Injection

**Figure 6:**
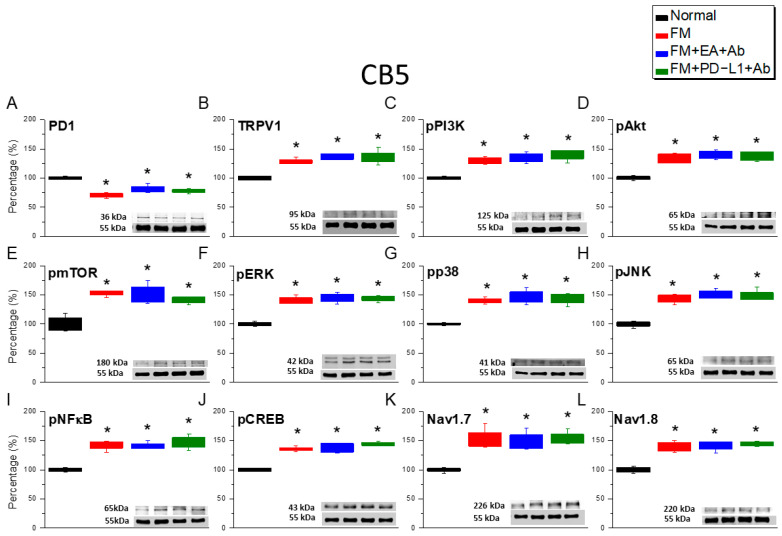
Protein levels of PD-1- and TRPV1-associated molecules in the CB5 region of mice. Western blotting contained four lanes: the normal, FM, FM + EA + Ab, FM + PD-L1 + Ab groups. (**A**) PD-1, (**B**) TRPV1, (**C**) pPI3K, (**D**) pAkt, (**E**) pmTOR, (**F**) pERK, (**G**) pp38, (**H**) pJNK, (**I**) pNFκB, (**J**) pCREB, (**K**) Na_v_1.7, and (**L**) Na_v_1.8. * *p* < 0.05 vs. normal group. *n* = 6. A one-way ANOVA test was used to determine the differences between the groups, followed by a post hoc Tukey test.

**Figure 7:**
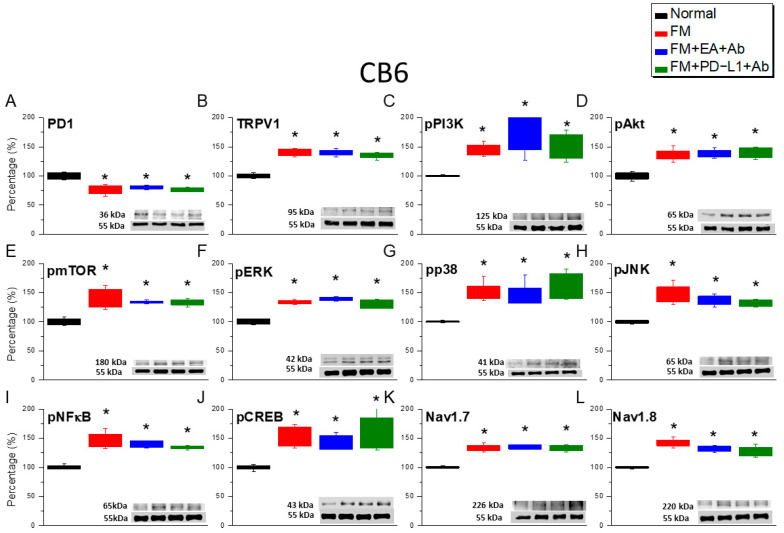
Protein levels of PD-1- and TRPV1-associated molecules in the CB6 region of mice. Western blotting contained four lanes: the normal, FM, FM + EA + Ab, FM + PD-L1 + Ab groups. (**A**) PD-1, (**B**) TRPV1, (**C**) pPI3K, (**D**) pAkt, (**E**) pmTOR, (**F**) pERK, (**G**) pp38, (**H**) pJNK, (**I**) pNFκB, (**J**) pCREB, (**K**) Na_v_1.7, and (**L**) Na_v_1.8. * *p* < 0.05 vs. normal group. *n* = 6. A one-way ANOVA test was used to determine the differences between the groups, followed by a post hoc Tukey test.

**Figure 8:**
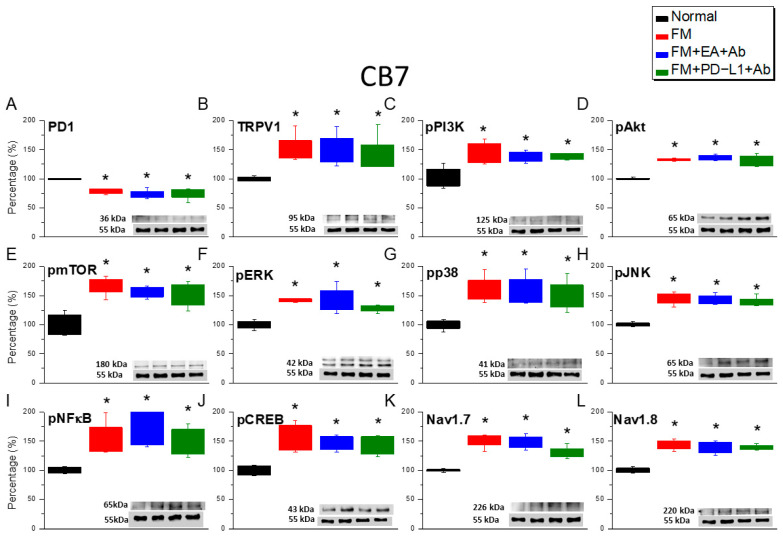
Protein expression of PD-1- and TRPV1-associated molecules in the CB7 region of mice. Western blotting contained four lanes: the normal, FM, FM + EA + Ab, FM + PD-L1 + Ab groups. (**A**) PD-1, (**B**) TRPV1, (**C**) pPI3K, (**D**) pAkt, (**E**) pmTOR, (**F**) pERK, (**G**) pp38, (**H**) pJNK, (**I**) pNFκB, (**J**) pCREB, (**K**) Na_v_1.7, and (**L**) Na_v_1.8. * *p* < 0.05 vs. normal group. *n* = 6. A one-way ANOVA test was used to determine the differences between the groups, followed by a post hoc Tukey test.

To further confirm the analgesic mechanism of EA and PD-L1 on nociceptive transduction in FM mice, we detected the protein expression levels of the aforementioned molecules in the CB5–7 regions. PD-1 levels in these regions significantly decreased in mice with ICS-induced FM (Figure 6A, Figure 7A and Figure 8A, red columns, *n* = 6). In mice administered with EA and concurrent ICV injection of a PD-L1-neutralizing antibody, we observed a decrease in PD-1 concentration, indicating that PD-L1/PD-1 is the mediator of EA analgesia (Figure 6, Figure 7 and Figure 8, blue columns, *n* = 6). Similar results were observed in mice simultaneously injected with PD-L1 and PD-L1 antibodies (Figure 6, Figure 7 and Figure 8, green columns, *n* = 6). Furthermore, ICS enhanced TRPV1 and pPI3K/pAkt/pmTOR signaling, as indicated by the elevated levels of mediators in the CB5–7 regions (Figure 6B–E, Figure 7B–E and Figure 8B–E, red columns, *n* = 6). Similar increases were observed in mice simultaneously receiving EA (Figure 6B–E, Figure 7B–E and Figure 8B–E, blue columns, *n* = 6) or PD-L1 injection and PD-L1 antibody injection (Figure 6B–E, Figure 7B–E and Figure 8B–E, green columns, *n* = 6). Following ICS induction, pERK/pp38/pJNK levels significantly increased in these regions (Figure 6F–H, Figure 7F–H and Figure 8F–H, red columns, *n* = 6). Similar results were observed in mice receiving EA (Figure 6F–H, Figure 7F–H and Figure 8F–H, blue columns, *n* = 6) or PD-L1 treatment (Figure 6F–H, Figure 7 F–H and Figure 8F–H, green columns, *n* = 6) concurrently with PD-L1 antibody injection. The expression of both pNFκB and pCREB proteins significantly increased in mice receiving ICS, EA, and PD-L1 injection (Figure 6I,J, Figure 7I,J and Figure 8I,J, *n* = 6) concurrently with PD-L1 antibody injection. Similar increases in expression were observed in Nav1.7 and Nav1.8 (Figure 6I,J, Figure 7I,J and Figure 8I,J, *n* = 6).

## 4. Discussion

The present study is the first to offer evidence that the modulatory effect of EA analgesia is mediated by PD-1 signaling in the cerebellum of FM mice. Our results identified a decrease in PD-1 receptor expression in the cerebellum after ICS-induced FM and a simultaneous increase in nociceptive TRPV1 signaling. In contrast, EA, PD-L1 injection, and *Trpv1* deletion exerted an antinociceptive effect which increased PD-1 expression in the cerebellum. Our data indicate that FM pain suppression is mediated by diminished TRPV1 signaling. In fact, the analgesic effects of EA and PD-L1 were abrogated by PD-L1-neutralizing antibody injection.

In recent work, researchers indicated that dysfunctional pain regulation is a trademark of FM and that exercise is a proper therapeutic remedy. They administered a 15-week mediation of strengthening exercises and suggested that FM patients had a decline in pain sensation, FM severity, and depression. A subgroup received functional magnetic resonance imaging in the course of counting thumbnail pressure pain stimulations. Their results showed a noteworthy therapeutic effect of exercise on the left dorsolateral prefrontal cortex and caudate. An increased neuronal connection from the caudate to the cerebellum was also indicated in the FM patients [25]. Kim et al. showed increased connections within the cerebellum of FM patients as well as more extensive connections in the frontal lobe compared to those in the normal group. In FM patients, via spectral partitioning, they also found a lower connection to the medial prefrontal cortex, and its gray matter volume was related to the severity of depression [26]. By using voxel-based morphometry (VBM) and diffusion tensor imaging, Mosch et al. revealed decreased gray matter (GM) sizes in the parahippocampal gyrus, dorsal anterior cingulate cortex (dACC), putamen, and left dorsolateral prefrontal cortex (DLPFC) in FM patients. In dissimilarity, significant augmented GM volume was detected in the cerebellum and thalamus [27]. Aster et al. found that, via resting-state functional magnetic resonance imaging (fMRI), fibromyalgia patients displayed abridged functional connectivity between the right midfrontal gyrus and the posterior cerebellum and right crus cerebellum [28]. Another article points out that when posterior lobe lesions affect lobules VI and VII (including Crus I, Crus II, and lobule VIIB), cognitive impairment occurs, weakening the cerebellum’s regulation of the cerebral association cortex [29].

Recently, by using VBM analysis, researchers revealed that the GM volume of the cerebellum was considerably enlarged in FM patients compared with patients without FM. The altered resting-state functional connectivity was abnormal in the cortico-striato-thalamo-cortical circuit in FM patients, with the lower function designating irregular reward, mood, decision, and motivation. The amplified GM volume in the cerebellum specified the contribution of the cerebellum in the irregular pain sensation in FM patients [30]. A recent paper reported that FM pain was accompanied by increased inflammatory mediators and toll-like receptor 4 (TLR4) signaling pathway expression in the mouse hypothalamus, periaqueductal gray, and cerebellum. Expression of TLR4 and downstream molecules including MyD88, TRAF6, pERK, pp38, and pJNK was increased in FM mice, an effect inhibited by 2 Hz EA treatment. The additional consequences indicated that activation of TLR4 by lipopolysaccharide considerably influenced FM pain and was then reversed by a TLR4 antagonist. This information demonstrates that EA analgesia functions via the TLR4 pathway [31].

PD-L1/PD-1 is a newly developed immunotherapy that can be used for many types of cancer, but there is a lack of evidence on its use in the treatment of pain. PD-1 receptors are generally expressed in brain neurons; it is unmet to develop PD-1 especially in analgesic peptides that had shown noteworthy beneficial effect with lower adverse properties. Recently, increasing PD-L1 to activate PD-1 was reported to moderate neuronal excitability and deliver antinociceptive effects, indicating its promising potential for pain relief [32,33,34]. It is worth noting that Zhao et al. developed a new small molecule, peptide H-20, that can activate PD-1 to achieve analgesic effects. Their in vitro results revealed that H-20 could bind to PD-1 with μM affinity to activate Src homology 2 domain-containing tyrosine phosphatase 1 (SHP-1) phosphorylation, which attenuated painful signals in the mice dorsal root ganglion (DRG). Pretreatment with H-20 successfully mitigated pain in normal mice. Intrathecal injection of H-20 resulted in significant anti-nociception in formalin-, acetic acid-, and CFA-initiated inflammatory pain models [35]. An excellent recent literature review pointed out that the PD-L1/PD-1 route in the DRG, sciatic nerve, and spinal cord delivered a chief role in several pain models. Exogenous PD-L1 activation actually attenuated pain symptoms in mice with bone cancer pain. The review also suggested that PD-L1 significantly increased the infiltration of macrophages into damaged nerves for pain relief. Additionally, augmentation of PD-L1/PD-1 in trigeminal ganglia neurons inhibits migraine pain [36].

## 5. Conclusions

**Figure 9:**
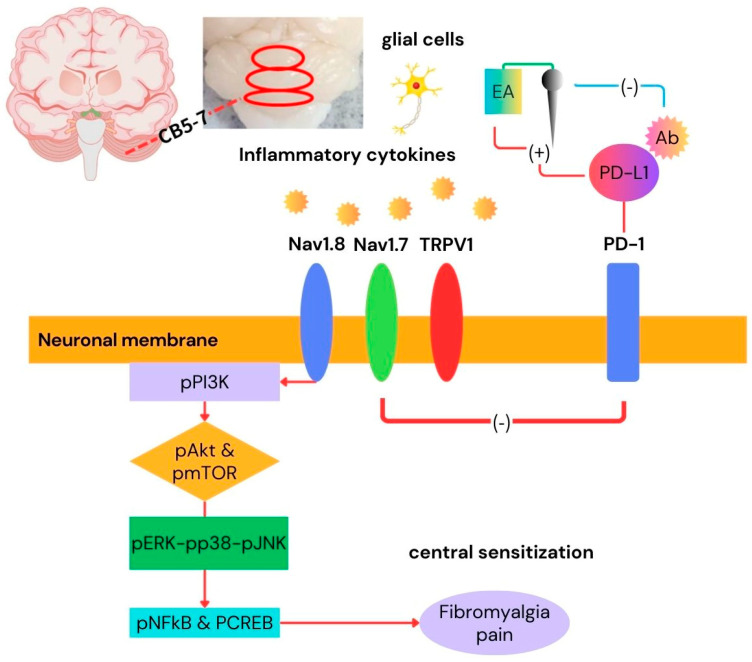
Graphic of EA in FM treatment via PD-1/TRPV1 and related molecules.

In sum, for the first time, we clarified that the effect of EA, PD-L1 injection, and *Trpv1* deletion in an FM pain model involved PD-1 and TRPV1 signaling. Interestingly, we validated that the antinociceptive effects of EA are mediated by the PD-1/TRPV1 signaling pathways. The current findings support the potential of EA as a therapeutic method for treating FM pain. Our results delivered their utility is importantly hindered in future clinical studies and serve EA as its therapeutic approaches for FM pain (Figure 9).

### 5.1. Main Findings and Implications

Our results mainly confirm the association between decreased PD-1 receptor expression and enhanced TRPV1-related pain kinase activity in the cerebellum of FM mice. EA and ICV injection of PD-L1 could alleviate FM pain by enhancing PD-1 receptor expression; similar results were observed in *Trpv1* knockout mice. ICV injection of PD-L1-neutralizing antibodies reversed these phenomena, confirming that the PD-1 receptor is the molecular mechanism of EA analgesia.

### 5.2. Study Strengths, Limitations, and Future Perspectives

EA precisely treats FM mice by activating PD-1 receptors. ICV administration of PD-L1-neutralizing antibodies reverses the analgesic effects of EA and PD-L1. These phenomena are attributed to the inhibition of TRPV pain-related molecular mechanisms by PD-1 receptors. Our study is limited by the fact that we only analyzed PD-1 receptors in the cerebellum of our model and cannot rule out the involvement of other receptors. Clinical trials are necessary to confirm our findings. We also have to examine more precise localization and cell-type-specific data beyond expression changes in the near future. This study only focused on the CB5-7 regions of the cerebellum, which is a shortcoming because it is insufficient to support claims of cerebellum specificity and translation.

## Data Availability

The data that support the findings of this study are available on request from the corresponding author. The data are not publicly available due to privacy or ethical restrictions.

## Abbreviations

The following abbreviations are used in this manuscript:

FM	Fibromyalgia
PD-1	Programmed cell death protein 1
EA	Electroacupuncture
ICS	Intermittent cold stress
TRPV1	Transient receptor potential vanilloid 1
ST36	Stomach 36
IL-1β	Interleukin 1β
TNF-α	Tumor necrosis factor-alpha
IFN-γ	Interferon gamma
VBM	Voxel-based morphometry
GM	Gray matter
dACC	Dorsal anterior cingulate cortex
DLPFC	Dorsolateral prefrontal cortex
TLR4	Toll-like receptor 4
MyD88	Myeloid differentiation primary response 88
TRAF6	Tumor necrosis factor receptor-associated factor 6
pERK	Phosphorylated extracellular signal-regulated kinase
pJNK	Phosphorylated c-jun n-terminal kinase
SHP-1	Src homology 2 domain-containing tyrosine phosphatase 1
DRG	Dorsal root ganglion
